# Supplementary material for: Melatonin-Mediated Regulation of Antioxidant Defense Enhances the Resistance of Tea Plants (Camellia sinensis L.) to Lead-Induced Stress
Source: Plants (Basel). 2025 May 9;14(10):1417. doi: 10.3390/plants14101417 (PMC12114777; doi:10.3390/plants14101417)
Supplement: Supplementary file 1 [file plants-14-01417-s001.zip › plants-3583606-supplementary.pdf]

Supplementary Material:

Melatonin-mediated Regulation of Antioxidant Defense Enhances the Resistance of Tea Plants (*Camellia sinensis L*) to Lead-induced Stress

Jianwu Li[1], Jiao Yang[1,2,#], Xin Liang[2], Shuping Zhan[1,2], Yixuan Bai[2]and Li Ruan[2,*]

1 College of Environment and Resources, Carbon Neutrality, Zhejiang A&F University, Hangzhou 311300, China
2 Institute of Sericulture and Tea, Zhejiang Academy of Agricultural Sciences, Hangzhou 310021, China
# These authors contributed equally to this work.
* Correspondence: ruanl@zaas.ac.cn

Table S1 The primer design for quantitative fluorescence PCR

**Primer names**	**Sequence(5′-3′)**
CsPAL-F	GAATGCCGGTCTTATCCACT
CsPAL-R	CGGTGAACACCTTGTCAAAC
CsC4H-F	CGAGAGGTTCTTGGAAGAGG
CsC4H-R	AGAATTGGCAGAGCAAGGAT
Cs4CL-F	CCTCCGGAACGACTGGATTA
Cs4CL-R	GCACGCACAACACCATATCT
CsCHS-F	GGCAATCAAAGAATGGGG
CsCHS-R	ATGGGCGAAGACCGAGT
CsCHI-F	CACAAAGAAGATTATGGGTGAAG
CsCHI-R	CAAACCTAGAAGTTGCCAAGAGT
CsF3H-F	CTACTCAAGATGGCCCGACAA
CsF3H-R	ACAACACCTCCAGCAACTTGC
CsDFR-F	ATTGGCAGAGAAAGCAGCAT
CsDFR-R	GTGATTAGGCTTGGTGGGAA
CsLAR-F	GTGATGGCAGTGTCAAAGCA
CsLAR-R	GGATGGTCGGAAATGAACCG
CsANR1-F	GCGAAGTTGATCCTCTCGTC
CsANR1-R	AACCACATCGTCAAGTGAACA
CsANR2-F	CATAGCCGGTTGTGACCTTG
CsANR2-R	ACAGAGGCTGCTGATGATGT
CsANS-F	GAATCTTCAAGGGTATGGGAGCA
CsANS-R	TGTAGTCGGTTGGTGTCTTGGGC
CsCDF1-F	TGCCTTCGCCATCTCCT
CsCDF1-R	GCTTCATCAGGGTCATCAATC
CsHMA5-F	CCATTGACCTATCCAGGAAAAC
CsHMA5-R	CAGCAAACAACACTAACCGAAG
CsZIP1-F	AGTCTGTCTTCCGTTGCTCG
CsZIP1-R	CGGGTTGGTCAGGTGGT
CsMRP3-F	GGAACCGAGATTCAAGGAGC
CsMRP3-R	CCAAGCGGAAGCATAGCC
CsMTP1-F	CAGAACCTTGCCTACTGTAATGG
CsMTP1-R	GCGACAAAACTTGAGATGGG
CsPDR12-F	TCAAGCAGTCCATCCACATC
CsPDR12-R	AGTCCGCAACTCCTTTCCT
